# Recommendations for breast imaging follow-up of women with a previous history of breast cancer: position paper from the Italian Group for Mammography Screening (GISMa) and the Italian College of Breast Radiologists (ICBR) by SIRM

**DOI:** 10.1007/s11547-016-0676-8

**Published:** 2016-09-06

**Authors:** Lauro Bucchi, Paolo Belli, Eva Benelli, Daniela Bernardi, Beniamino Brancato, Massimo Calabrese, Luca A. Carbonaro, Francesca Caumo, Beatrice Cavallo-Marincola, Paola Clauser, Chiara Fedato, Alfonso Frigerio, Vania Galli, Livia Giordano, Paola Golinelli, Giovanna Mariscotti, Laura Martincich, Stefania Montemezzi, Doralba Morrone, Carlo Naldoni, Adriana Paduos, Pietro Panizza, Federica Pediconi, Fiammetta Querci, Antonio Rizzo, Gianni Saguatti, Alberto Tagliafico, Rubina M. Trimboli, Chiara Zuiani, Francesco Sardanelli

**Affiliations:** 1 Romagna Cancer Registry, Romagna Cancer Institute (IRST) IRCCS, via Piero Maroncelli, 40, 47014 Meldola, Forlì Italy; 2 Dipartimento di Scienze Radiologiche, Università Cattolica del Sacro Cuore, Largo Agostino Gemelli, 8, 0168 Rome, Italy; 3 Zadig Scientific Communication Agency, via Arezzo 21, 00161 Rome, Italy; 4 Dipartimento di Radiologia, U.O. Senologia Clinica e Screening Mammografico, APSS, Centro per i Servizi Sanitari, Pal. C, viale Verona, 38123 Trento, Italy; 5 Struttura Complessa di Senologia Clinica, Istituto per lo Studio e la Prevenzione Oncologica (ISPO), Via Cosimo il Vecchio 2, 50139 Florence, Italy; 6 UOC Senologia Diagnostica, IRCCS AOU San Martino-IST, Largo Rosanna Benzi 10, 16132 Genoa, Italy; 7 Unit of Radiology, Research Hospital (IRCCS) Policlinico San Donato, Via Morandi 30, San Donato Milanese, 20097 Milan, Italy; 8 UOSD Breast Unit ULSS20, Piazza Lambranzi 1, 37142 Verona, Italy; 9 Dipartimento di Scienze Radiologiche, Oncologiche ed Anatomo-patologiche, Policlinico Umberto I, Sapienza Università di Roma, Viale Regina Elena 324, 00161 Rome, Italy; 10 Department of Biomedical Imaging and Image-guided Therapy, Division of Molecular and Gender Imaging, Medical University of Vienna/General Hospital Vienna, Waehringer Guertel 18-20, 1090 Verona, Austria; 11 Institute of Radiology, University of Udine, P.le S. M. della Misericordia 15, 33100 Udine, Italy; 12 Regional Screening Coordinating Centre, Veneto Region, Venice, Italy; 13 Regional Reference Centre for Breast Cancer Screening, Turin, Italy; 14 Mammography Screening Centre, Local Health Authority, Modena, Italy; 15 Epidemiology Unit, Centre for Cancer Prevention, Turin, Italy; 16 Medical Physics Service, Local Health Authority, Modena, Italy; 17 Dipartimento di Diagnostica per Immagini, Radiologia 1U, Università di Torino, A. O. U. Città della Salute e della Scienza di Torino, Via Genova 3, 10126 Turin, Italy; 18 U.O. Radiodiagnostica, Candiolo Cancer Institute-FPO, IRCCS, Str. Prov. 142, km 3.95, I, 10060 Candiolo Turin, Italy; 19 DAI Patologia e Diagnostica, Azienda Ospedaliera Universitaria Integrata, P.le A. Stefani 1, 37126 Verona, Italy; 20 Department of Health, Emilia-Romagna Region, Bologna, Italy; 21 U.O. Radiologia Senologica, IRCCS Ospedale San Raffaele, Via Olgettina 60, 20132 Milan, Italy; 22 Department of Prevention, Screening Centre, Local Health Authority, Sassari, Italy; 23 Pathology Department, Local Health Authority, Asolo, Italy; 24 Senology Unit, Local Health Authority, Bologna, Italy; 25 Department of Experimental Medicine, DIMES, Institute of Anatomy, University of Genova, Via de Toni 14, 16132 Genoa, Italy; 26 Department of Biomedical Sciences for Health, Università degli Studi di Milano, Via Morandi 30, San Donato Milanese, 20097 Milan, Italy

**Keywords:** Breast cancer, Follow-up, Mammography, Screening, Survivorship care

## Abstract

Women who were previously treated for breast cancer (BC) are an important particular subgroup of women at intermediate BC risk. Their breast follow-up should be planned taking in consideration a 1.0–1.5 % annual rate of loco-regional recurrences and new ipsilateral or contralateral BCs during 15–20 years, and be based on a regional/district invitation system. This activity should be carried out by a Department of Radiology integrating screening and diagnostics in the context of a Breast Unit. We recommend the adoption of protocols dedicated to women previously treated for BC, with a clear definition of responsibilities, methods for invitation, site(s) of visits, methods for clinical and radiological evaluation, follow-up duration, role and function of family doctors and specialists. These women will be invited to get a mammogram in dedicated sessions starting from the year after the end of treatment. The planned follow-up duration will be at least 10 years and will be defined on the basis of patient’s age and preferences, taking into consideration organizational matters. Special agreements can be defined in the case of women who have their follow-up planned at other qualified centers. Dedicated screening sessions should include: evaluation of familial/personal history (if previously not done) for identifying high-risk conditions which could indicate a different screening strategy; immediate evaluation of mammograms by one or, when possible, two breast radiologists with possible addition of supplemental mammographic views, digital breast tomosynthesis, clinical breast examination, breast ultrasound; and prompt planning of possible further workup. Results of these screening sessions should be set apart from those of general female population screening and presented in dedicated reports. The following research issues are suggested: further risk stratification and effectiveness of follow-up protocols differentiated also for BC pathologic subtype and molecular classification, and evaluation of different models of survivorship care, also in terms of cost-effectiveness.

## Introduction

This position paper on recommendations for breast imaging follow-up of women with a previous history of breast cancer (BC) is the result of an agreement between the Italian Group for Mammography Screening (GISMa) and the Italian College of Breast Radiologists (ICBR) by the Italian Society of Medical Radiology (SIRM). The decision to provide this paper was taken at the end of a joint GISMa/ICBR-SIRM workshop held in Reggio Emilia on May 8, 2015 in the context of GISMa annual meeting. The text has been approved by the GISMa Coordination Board and the ICBR-SIRM Board of Directors.

## Background

In Italy, according to the Italian Association of Cancer Registries (AIRTUM) [1], female BC survivors in 2015 were about 693,000, equal to 2.2 % of female population and to 42 % of overall cancer prevalence among women.

After conservative treatment, a peak of ipsilateral true loco-regional recurrences is observed during the first 5 years, in particular the first 2 years; thereafter, this risk is progressively fading [2]. This temporal trend is similar to that of distant metastases [3]. Conversely, the risk of a new primary contralateral BC and its cumulative incidence increase over time: the majority of these events are observed after the first 5 follow-up years [4, 5]. This risk profile can be applied also to women who underwent unilateral mastectomy. Among ipsilateral recurrences too, the new primary BCs (about 50 % of recurrences) have a later onset [6]. Thus, the overall incidence of true loco-regional recurrences and of new primary BCs shows a steady annual rate of 1.0–1.5 % during 15–20 years [7], resulting in a continuous increase of the cumulative incidence [4, 8].

Studies reporting higher relapse rates during the first 3 years [3] or 3–5 years [2, 9] included true loco-regional recurrences, new primary ipsilateral BCs, and distant metastases but excluded the new primary contralateral BCs. As a consequence, the authors observed an early peak of events, which biased guidelines and recommendations issued by medical societies and governmental agencies.

Breast imaging follow-up should be planned accounting for an overall annual rate of loco-regional recurrences and new primary ipsilateral or contralateral BCs equal to 1.0–1.5 % during 15–20 years. A more intensive breast surveillance during the first 3–5 years and a subsequent less intensive surveillance have no rational bases [8, 10]. *Women with a previous BC history should be considered as an important particular subset of women with an intermediate BC risk*, lower than that of BRCA or p53 mutation carriers and higher than that of women with neither personal nor familial BC history, or with only sporadic BCs among their relatives [11].

No evidence is available from randomized controlled trials on effectiveness of breast imaging follow-up in terms of mortality reduction. Observational studies comparing women undergoing different follow-up strategies including annual or biannual mammography versus no follow-up reported variable mortality reductions or an increased long-term survival independent of lead time [12–15]. No studies reported a comparison in terms of effectiveness between clinical/radiological follow-up in a *diagnostic* context versus invitation to a population-based organized screening program [16]; moreover, no studies reported on the effectiveness of annual versus biannual follow-up mammography [15].

The value of clinical breast examination (CBE) for women with a previous personal BC history is uncertain. Technical development of mammography (in particular from film-screen to digital [17]) and quality assurance programs reduced the rate of relapses diagnosed with CBE to only 15 % [10]. Notably, the relapses diagnosed with CBE are associated with a shorter survival than those diagnosed with mammography [10]. However, CBE still has a role in diagnosing axillary recurrences as well as metastases at supraclavicular region or thoracic wall and for the examination of the surgical scar. Moreover, CBE is a relevant occasion for getting information about the patient’s personal and family history in the context of a *cancer survivorship care,* paying also attention to psychological issues.

No studies demonstrated a survival benefit from an earlier diagnosis of asymptomatic breast recurrence/relapse using magnetic resonance imaging (MRI) [11, 18] or breast ultrasound [11].

International guidelines differ in many important issues of breast follow-up of women with a previous BC history: frequency of clinical mammography and possible CBE; (re)inclusion in population-based organized screening programs and timing for this inclusion; frequency of screening mammography also considering the individual risk profile [19–23]. As mentioned above, some medical societies and governmental bodies recommend a higher frequency of mammography and CBE during the first 3–5 years [19, 22, 23], even though the temporal trend of the true loco-regional recurrences and new primary ipsilateral and contralateral tumors does not support this recommendation.

For population-based organized screening programs, the management of women previously treated for BC is an open issue. These women are excluded from screening programs in many countries [24–26] but are included in other countries [27–29]. Different policies are adopted at the regional level in some countries such as in Canada, where some regional programs invite women previously treated for BC to screening mammography even though the data are excluded from standard statistical analysis of the programs’ performance [30].

In Italy, the GISMa has so far recommended to exclude from invitation only those women who are certainly followed up at clinical centers. In fact, many regional programs exclude from invitation all women previously treated for BC in a systematic manner. This practice is a matter for debate [31, 32] because only half of health care districts are served by an active follow-up service and only half of programs (re)invite these women after a variable time since the BC event (2015 GISMa survey, *unpublished data*).

Follow-up protocols also vary greatly in the diagnostic/clinical context, given the lack of reliable effectiveness studies [33]. The screening practice too, whether organized or spontaneous, contributes to this variability: screen-detected cancers amplify the heterogeneity of biological profiles of BCs as a length bias effect [34]. Of note, the use of different follow-up protocols according to the individual biological tumor profile is practice commonly performed but not validated. Only one model for risk estimation of annual loco-regional recurrence has been developed [35].

The definition of breast imaging follow-up protocols for women previously treated for BC is necessarily influenced by the debate on organizational issues for BC care and, in a more general perspective, on follow-up in cancer patients. On the one side, the Breast Unit model is increasingly applied as a territorial facility for multidisciplinary breast care, formally addressed by the European Parliament in 2006 to support its general application in the European Union from 2016 [36] and recently adopted in Italy by the *Conferenza Stato*-*Regioni* 1 [37]. On the other side, the concept of *cancer survivorship care* (i.e., the survival status as a phase of a continued cancer care) [38] is progressively adopted by health systems. This phase starts at the end of primary treatment and should offer specialized comprehensive answers to the person’s needs: information on life styles; management of comorbidities and of side effects of treatments; identification of long-term physical, psychological, and social effects of the disease and disease-related treatments; identification of disease effects on families; evaluation of survival quality. The complexity of these issues makes the Breast Unit the most appropriate facility for offering a comprehensive follow-up service to these women.

However, we should not use the concept of *cancer survivorship care* to change the condition of women previously treated for BC into long-term survivors, taking into consideration also the economic impact of long-term intensive surveillance protocols. If we really evaluate the psychological impact of a BC diagnosis and of the subsequent recovery, after a first phase of intensive relationship of the patient with the facility where the treatment has been done, it appears to be important that we consider these women not as *survivors* but as an important subset of women at increased risk for BC. As a consequence, these women should be included into invitation lists of screening programs, with a frequency and modalities adapted to the increased risk level. This strategy is derived from a comprehensive evaluation, not only from considering economic cost of prolonged intensive surveillance in the clinical setting.

In this perspective, follow-up of women previously treated for BC should be active, i.e., using a centralized invitation system, and have a territorial basis as an institutional activity dedicated to all prevalent BC cases. This approach should also protect all women previously treated for BC, providing them with a planned access to mammography also when, in the case of spontaneous surveillance, for different reasons such as other relevant familial events and psychological removal of the BC occurrence, the mammogram would be deferred or not performed at all [31]. Moreover, in this way we could also offer a protection to those women who are treated for BC at major hospitals or cancer centers but live in different regions, as frequently occurs in Italy.

The general perspective is that screening and clinical breast imaging will be offered by the same Department of Radiology, in the context of a Breast Unit. The current implementation of the Breast Units in Italy and their connection with screening programs have to be considered a transition process, which is relevant for the following recommendations.

## Recommendations

The Breast Unit should define a dedicated follow-up protocol for women already treated for BC. This protocol should describe responsibilities, invitation, facilities, radiological and clinical procedures, and duration of the follow-up. Role and function of family doctors and BC specialists should be also defined.

Women already treated for any BC (screen-detected BCs from organized population-based programs, interval cancers, BC cases diagnosed outside screening programs in asymptomatic or symptomatic women) should not be excluded from screening invitation lists. Screening programs, in the context of the Breast Unit, should invite for a mammogram those women treated for BC who reside in the catchment area, independently of the age at diagnosis and current woman’s age, at least up to 74 years of age, starting from the year following treatment. These women should be examined in dedicated sessions (for cases of bilateral mastectomy, see below). The follow-up duration should not be shorter than 10 years and will be defined on the basis of woman’s age and local considerations.

If another service or center has included one of these woman in a follow-up program (e.g., the cancer center where the BC was treated) and informs the Breast Unit of this, the Breast Unit should be open to make an agreement with the other service for excluding or including that woman in the invitation list. If the invited woman declares to prefer to be followed by another service, the Breast Unit should notify to this service the woman’s willingness and plan not to invite her for the time indicated by the other center.

The dedicated screening sessions should be organized according to a defined protocol, here summarized as an indicative reference:

1. investigation of personal/familial history (if not already done) to identify high-risk conditions for which a different screening strategy should be adopted;
2. CBE and possible breast ultrasound performed by a breast radiologist in the case of bilateral mastectomy;
3. immediate mammogram reading by a breast radiologist (single reading) or, when possible, by two breast radiologists (blinded double reading);
4. immediate performance of supplemental investigations (CBE, other mammographic views, tomosynthesis and/or breast ultrasound, and, whenever possible, needle sampling);
5. fast scheduling (preferably within ten working days) of further workup including, if indicated, MRI, according to guidelines [17, 18, 39].

The key point of the protocol should be the immediate communication of the results of the session to the woman, possibly with personal interaction between the breast radiologist and the woman and with a written report.

In the case of single reading to reach a diagnostic conclusion to be immediately communicated to the woman, the second reading is waived considering that the comprehensive clinical evaluation can also include the above-mentioned supplemental techniques. However, although psychological issues would advise against a delayed second reading, this option can be considered if a high-quality interaction with the woman is undertaken.^2^

The Breast Unit should coordinate as much as possible the breast follow-up rounds and other follow-up visits, trying to allow the woman to have all visits on the same day.

Considering the special features of these dedicated sessions, costs should be specifically evaluated. Payments should be differentiated from those of screening mammography offered to the general female population in the absence of BC history.

Data about screening results of women already treated for BC should be hived off from those regarding the general female population invited to screening and should be presented in dedicated annual reports, as happens in other countries [30]. In particular, among the other usual indicators, attention should be paid to: crude and adjusted response rate; prevalent BC cases; quantity and quality of the offered service; and women’s satisfaction evaluation. Results from both single reading protocols and blinded double reading protocols (either with immediate or delayed second reading) will undergo an evaluation by the Breast Unit, paying careful attention to: ipsilateral or contralateral relapse diagnosed at stage T2 or greater; interval cancer analysis; in the case of delayed second reading, additional recall rate due to the second reading and compliance rate in the following rounds.

From the viewpoint of the economic impact, apart from the already mentioned specific reimbursement negotiations, the authors evaluate that the cost cannot be superior to that implied by the current practice of annual CBE and mammography with or without breast ultrasound, commonly performed in women with previous BC history. However, the implementation of such a protocol will require time and specific organizational choices, also considering the inhomogeneity of Breast Unit organization in the Italian regions.

Follow-up of women already treated for BC should be considered as a strategic area of BC research. In particular, we suggest the following research lines:

1. value of double reading (immediate or delayed) when screening women already treated for BC, with studies specifically designed (blinded sequential reading with or without arbitration, randomized controlled trials) and including both ipsilateral and contralateral cases;
2. usefulness of a further risk stratification and effectiveness of different follow-up protocols, which may be tuned to pathologic and biomolecular features of the first BC and patient’s age and history and may be evaluated using surrogate endpoints;
3. value of different models for survivorship care [40], also in terms of cost-effectiveness.

Starting 3 years after the publication of these recommendations, GISMa and ICBR/SIRM will evaluate the degree of their implementation in Italy through surveys among their members.

## Footnotes

1 In official Italian documents, the *Breast Unit* denomination has been translated into *Centro di Senologia*.

2 When the delayed second reading is applied, the breast radiologist who works as first reader, in the case of suspicious finding(s), will perform all suitable workup, while, in the case of negative evaluation, will give the woman the negative report in order to reassure her. However, at the same time, the radiologist will inform the woman that her mammograms will be read also in a delayed time by second reader (according the usual protocol for screening mammography) who occasionally could recall the woman for further workup. This information will be also included in the negative written report.
